# The Economic Burden of Gestational Diabetes and Body Mass Index Changes Between Pregnancies: A Retrospective Cohort Study

**DOI:** 10.1111/1471-0528.70208

**Published:** 2026-03-10

**Authors:** Rashidul Alam Mahumud, Glynis Pauline Ross, Adam Mackie, Rachael L. Morton, Kirsten I. Black

**Affiliations:** ^1^ NHMRC Clinical Trials Centre, Faculty of Medicine and Health The University of Sydney Sydney New South Wales Australia; ^2^ Department of Endocrinology Royal Prince Alfred Hospital, Sydney Local Health District Sydney New South Wales Australia; ^3^ Central Clinical School, Faculty of Medicine and Health, and Charles Perkins Centre University of Sydney Sydney New South Wales Australia; ^4^ Department of Women's Health, Neonatology and Paediatrics Royal Prince Alfred Hospital, Sydney Local Health District Sydney New South Wales Australia

**Keywords:** gestational diabetes mellitus, health care costs, length of stay, obesity, overweight

## Abstract

**Objective:**

To determine associations between gestational diabetes mellitus (GDM) and body mass index (BMI) change between consecutive pregnancies, and hospital length of stay (LOS) and hospital costs.

**Design:**

Retrospective cohort study.

**Setting:**

Two maternity hospitals, New South Wales, Australia.

**Population:**

Women with two most recent singleton births between September 2011 and April 2019.

**Methods:**

Multivariable generalised linear models assessed association between GDM status (none, first only, second only, or both pregnancies) and BMI change (loss: < −1 kg/m^2^; stable: −1 to < 1; small gain: 1 to < 2; medium gain: 2 to < 4; or large gain: ≥ 4) in relation to second‐birth LOS and hospital birthing costs. Costs were calculated using DRG codes and valued in 2024 AUD.

**Main Outcome Measures:**

Hospital LOS and hospital costs for the second birth.

**Results:**

Of 11 157 women, 5% had GDM only in the first pregnancy, 9% only in the second and 8% in both. For the second birth, women with GDM in both pregnancies stayed 0.91 days longer (3.71 vs. 2.79 days) and incurred AU$1960 higher costs (AU$14680 vs. AU$12720) than those without GDM in either pregnancy. Adjusted models indicated GDM in both pregnancies was associated with a 21% longer LOS (95% CI: 16%–26%, *p* < 0.001) and 9% higher costs (7%–11%, *p* < 0.001). Large BMI gain (≥ 4 kg/m^2^) was associated with a 9% longer LOS (4%–14%, *p* < 0.001). Women with both GDM (both pregnancies) and large BMI gain had a 23% longer LOS (12%–36%, *p* < 0.001) and 9% higher costs (4%–15%, *p* < 0.001).

**Conclusion:**

GDM and interpregnancy BMI gain were associated with increased hospital stay and higher birthing‐related hospital costs for the second birth. These findings underscore the importance of preventive strategies and supportive public health policies to reduce the burden of GDM and high BMI gain. Approaches such as nutritional counselling, lifestyle modification and physical activity programs are likely to support women planning a subsequent pregnancy after their index birth (first pregnancy or first pregnancy with GDM diagnosis).

## 1 Introduction

Rising rates of maternal overweight and obesity have been associated with adverse pregnancy and birth outcomes, including hypertensive disorders, induction of labour, operative delivery, postpartum haemorrhage and fetal complications such as large‐for‐gestational‐age babies, congenital malformations, stillbirth and shoulder dystocia [1, 2, 3, 4]. High body mass index (BMI) also increases the risk of gestational diabetes mellitus (GDM), defined as glucose intolerance diagnosed for the first time in pregnancy. GDM complicates approximately 1 in 7 pregnancies worldwide [5]. The repercussions of GDM are similar to maternal overweight and obesity [6]; both have intergenerational effects, increasing risk for childhood obesity [7] or later metabolic disease [8]. Mothers who develop GDM have an increased likelihood of developing future type 2 diabetes and cardiovascular disease [9].

The economic burden of GDM and high BMI in pregnancy has been documented in several settings, including Australia and the UK, with higher hospital utilisation and childbirth‐related costs reported in single‐pregnancy analyses [10, 11, 12, 13, 14]. However, single‐pregnancy cost estimates do not capture how a woman's risk profile changes between pregnancies (e.g., incident, persistent, or resolved GDM) or how interpregnancy BMI change may modify hospital length of stay (LOS) and costs for the subsequent birth. Understanding this ‘second‐birth’ perspective is important because the interpregnancy period represents a window for clinical interventions to prevent and modify these risks that could influence outcomes and healthcare use in the subsequent pregnancy.

Several cohort studies have documented the relationship between weight gain and pregnancy on the risk of developing GDM. A Norwegian cohort of 24 198 mothers reported that BMI gain increased GDM risk, while overweight women who had a weight reduction ≥ 2 BMI units also reduced their chance of developing GDM [8]. A Californian study similarly found higher GDM risk with BMI gain and reduced GDM risk among those who were overweight or obese who had weight loss [9]. In our Australian cohort of women with at least two consecutive singleton births between 2010 and 2017, we found that BMI reduction between births among women without prior GDM was associated with a 36% lower subsequent risk. Conversely, BMI gain ≥ 4 kg/m^2^ markedly increased risk [15]. We also estimated that reducing BMI from obesity to overweight could have prevented 14.2% of GDM diagnoses between 2010 and 2014 [16].

Despite this evidence on clinical risk trajectories, less is known about how these between‐pregnancy changes translate into hospital resource use and costs for a subsequent birth. Prior studies suggest that overweight and obese women with GDM incur a 23% higher hospital costs within a single pregnancy, compared with women with normal BMI [10, 11]. However, to our knowledge, no studies have examined hospital stay and birth‐related hospital costs at the subsequent (second) birth using a within‐woman, linked data approach that incorporates longitudinal GDM status and interpregnancy BMI change. This approach provides insights beyond single‐pregnancy analyses by capturing within‐woman transitions in risk and identifying subgroups in whom excess utilisation and costs are concentrated; information that can better inform targeted interpregnancy monitoring and prevention.

Given the potential for BMI change to improve or worsen the risk of GDM between pregnancies, we explored how hospital LOS and costs were impacted. Specifically, we investigated how GDM diagnosis and BMI change between two consecutive births are associated hospital LOS and hospital birthing costs for the second birth in New South Wales, Australia. We hypothesised that: (1) women without GDM will have lower hospital LOS and hospital birthing costs compared with women diagnosed with GDM; and (2) women with stable or decreased BMI will have lower LOS and hospital birthing costs for their second birth than women with BMI gain.

## 2 Methods

### 2.1 Study Design and Setting

A retrospective cohort study was conducted in New South Wales (NSW), Australia. We used electronic medical record data from women who delivered consecutive singleton births at the Royal Prince Alfred Hospital or Canterbury Hospital in the Sydney Local Health District (SLHD), NSW, Australia. The SLHD accounts for over 7693 births annually, representing more than 8% of all births in NSW in 2019 [17]. This included women who had a first‐ever (index) birth, regardless of GDM diagnosis status, between October 2010 and December 2017, and a subsequent (second) birth between September 2011 and April 2019. The ‘*index birth’* was defined as the earlier of the two most recent births; ‘*subsequent birth’* was the next birth following the index. We used unique person‐numbers and dates to identify the most recent two singleton births for each woman.

### 2.2 Participants

All women meeting the following criteria were included: (i) at least two consecutive singleton births of at least 20 weeks' gestation, at one of two maternity hospitals in the SLHD; (ii) medical record data for both births were available including Australian Refined Diagnosis Related Group (AR‐DRG) codes for birthing, including labour ward, delivery room, birthing suite, operating theatre caesarean section, other surgery and postnatal ward admissions. Records of pregnant women who did not meet these criteria were excluded from the analysis.

### 2.3 Outcome Measures

Maternal hospital length of stay (LOS) in days for birthing, and associated birthing‐related admitted hospital costs valued in 2024 Australian dollars. Further details of the LOS and cost calculations are provided below.

#### 2.3.1 Hospital Length of Stay (LOS)

Maternal hospital LOS for birthing was defined as the number of days from hospital admission to discharge for the delivery admission. Therefore, LOS was calculated as the discharge period (date and time) minus the hospital admission date and time within the same admitted birth episode for each birth separately.

#### 2.3.2 Maternal Hospitalisation Birthing Costs

Maternal birthing costs were calculated using the AR‐DRG classification related to the admitted maternal birth episode. Costs were estimated by applying 2024–25 National Efficient Price (NEP) weights to each maternal and neonatal AR‐DRG classification, using the NEP Determination 2024–25 base price per weighted hospital stay. National cost weights as determined by the Independent Health and Aged Care Authority Pricing for each AR‐DRG code were then applied to determine admitted acute hospital costs [18]. Each woman's maternal birthing cost comprised the total cost of an admitted acute hospital episode associated with the delivery, and included neonatal admitted costs where the neonate was also admitted during the same birth episode. Subsequent post‐discharge maternal or neonatal hospitalisations were not included. Costs were calculated for the initial and subsequent births separately. Costs were reported as means and rounded to whole AU$, using the 2024 reference year [18].

### 2.4 Main Exposure Variables

#### 2.4.1 Gestational Diabetes Mellitus (GDM)

Clinical diagnoses at the time of admission to hospital were coded using the 10th edition of the International Classification of Diseases–Australian modification (ICD‐10‐AM) including (GDM) in the birth admission (ICD‐10‐AM O24.4). During the study period, Australian diagnostic practice for GDM followed the Australasian Diabetes in Pregnancy Society (ADIPS) guidance, with criteria updated on 1 January 2015 from earlier ADIPS 1991/1998 recommendations. Prior to 2015, ADIPS 1991/1998 criteria generally used a two‐step process: universal screening/testing was recommended; women at high risk proceeded directly to a diagnostic 75‐g oral glucose tolerance test (OGTT), while lower‐risk women underwent a 50‐g glucose challenge test (GCT) with a 1‐h threshold of ≥ 7.8 mmol/L triggering a diagnostic 75‐g OGTT. Diagnosis of GDM on the 75‐g OGTT was made if one or more values met or exceeded the following cut‐offs: fasting ≥ 5.5 mmol/L and/or 2‐h ≥ 8.0 mmol/L. After 1 January 2015, following adoption of the updated ADIPS criteria, a one‐step 75‐g OGTT at 24–28 weeks (earlier if high‐risk) was recommended [10], with GDM diagnosed if one or more thresholds were met: fasting ≥ 5.1 mmol/L; 1 h ≥ 10.0 mmol/L; or 2 h ≥ 8.5 mmol/L after a 75 g glucose load. Because GDM status was identified using clinician‐assigned ICD‐10‐AM diagnosis codes recorded at the time of birth admission, classification reflects contemporaneous national clinical practice; therefore, reclassification or sensitivity analyses by diagnostic era were not undertaken.

We derived a new variable to assess the association of maternal GDM exposure on hospital LOS and hospital costs for maternal birthing admissions, a longitudinal GDM exposure variable was constructed using information from both the first and second pregnancies. Women were categorised into four mutually exclusive groups based on GDM status during consecutive pregnancies: GDM‐G_1_: experienced no GDM in either pregnancy (reference group); GDM‐G_2_: experienced no GDM in 1st pregnancy but GDM in 2nd pregnancy; GDM‐G_3_: experienced GDM in 1st pregnancy but no GDM in 2nd pregnancy; or GDM‐G_4_: experienced GDM in both pregnancies.

#### 2.4.2 Body Mass Index (BMI) Categories

BMI was defined as weight in kilograms divided by height in metres squared (kg/m^2^) and was calculated using self‐reported pre‐pregnancy weight and height recorded at the first antenatal visit. BMI was categorised according to WHO guidelines: underweight (< 18.5 kg/m^2^), normal weight (18.5–24.9 kg/m^2^), overweight (25–29.9 kg/m^2^) and obese (≥ 30 kg/m^2^) [15]. Change in BMI was calculated as the difference between pre‐pregnancy BMI for the second birth minus the pre‐pregnancy BMI in the first (index) birth, thereby capturing interpregnancy BMI change. Change in BMI was grouped into five categories: group‐a weight loss (< −1 kg/m^2^); group b stable weight (−1 to < 1 kg/m^2^) (reference group); group‐c weight gain (1 to < 2 kg/m^2^); group‐d weight gain (2 to < 4 kg/m^2^); and group‐e weight gain (≥ 4 kg/m^2^) [19].

### 2.5 Covariates

Relevant covariates with a known or hypothesised association with the exposure variables (i.e., GDM diagnosis, or change in BMI category) or outcomes of interest (hospital LOS and costs) were identified from published literature [10, 12, 13, 15, 20, 21, 22, 23, 24, 25]. Several covariates were included in regression models to answer each research question as outlined in the statistical considerations below. Mode of delivery was recorded as a four‐category variable: vaginal normal birth, instrumental delivery, caesarean section‐emergency, or caesarean section‐elective. Maternal age was grouped as ‘16–24 years’, ‘25–29’ years, ‘30–34 years’ or ‘≥ 35 years.’ Gestational age in weeks was categorised as ‘pre‐term: < 37 weeks’ gestation’, ‘term: 37–41 weeks' or ‘post‐term: ≥ 42 weeks'. Pregnancy‐specific covariates, including mode of delivery, maternal age and gestational age, were defined using information from the second birth only, as these characteristics cannot be meaningfully combined across pregnancies. Interpregnancy (first birth to second birth) intervals in months were categorised into six groups (< 6 months, 6–11 months, 12–17 months, 18–23 months, 24–59 months, or ≥ 60 months). This variable was defined as a composite measure using information from both the first and second births. Finally, socio‐economic disadvantage was obtained by the index of relative socio‐economic disadvantage and categorised into five groups (quintile 1 = most disadvantaged area, quintile 2, quintile 3, quintile 4, or quintile 5 = least disadvantaged area), with area‐level disadvantage assigned based on maternal residence at the time of the second birth.

### 2.6 Statistical Considerations

In the descriptive analyses, participant characteristics were summarised using frequencies (*n*, %) for categorical variables, mean (standard deviation, SD) for continuous data accounting for the skewed distribution of hospitalisation length of stay and maternal birthing costs (Table S1 and Figure S1). A univariate generalised linear model (GLM) was used to assess differences in hospital LOS or maternal birthing costs by GDM group and change in BMI category [26]. Primary regression analyses were conducted using a complete‐ case analysis. For missing data, multiple imputation was applied by chained equations [27] as part of sensitivity analyses to assess the robustness of results to missing BMI data. The imputation model included BMI as the imputed variables, with maternal age, gestational age category and delivery type as covariates. Linear regression was used under the assumption of a monotone missing pattern. A total of 20 imputed datasets were generated for each BMI variable using the ‘mi impute chained’ command. A fixed random number seed (rseed(12345)) was applied to ensure the imputation process was reproducible. Model estimates across imputed datasets were combined using Rubin's rules to produce pooled estimates and robust standard errors [28]. The multicollinearity problem was tested using Variance Inflation Factor (VIF). The decision rules applied were: (i) no evidence of multicollinearity when 0 < VIF < 5, (ii) moderate multicollinearity when 5 ≤ VIF ≤ 10 and (iii) high multicollinearity when VIF > 10 [29].

For both univariate and multivariable analyses, the choice of estimation approach was informed by the nature of the outcome variables under consideration in each model [30, 31]. Unadjusted and adjusted associations between hospital LOS; hospitalisation birthing costs, and the main exposure variables were estimated using GLM with a gamma distribution with log‐link function, and robust estimates of variance. The choice of link function was informed by the Pregibon link test [31], while the appropriate distribution family was determined using the Modifed Park's test [30]. The lower‐risk category of covariates was used as the referent group. Sensitivity analyses were undertaken to test the robustness of model results using multivariable GLMs with imputed BMI change values to examine hospital LOS and maternal hospital birthing costs in the second birth. Additional robustness checks were conducted using a non‐parametric bootstrapping approach 10 000 replications applied to models with imputed BMI change values. This, in turn, was used to adjust for the effects of other potential confounders. We explored interaction effects in the regression models. Interaction effects between longitudinal GDM status and interpregnancy BMI change categories were examined by including multiplicative interaction terms in the regression models. These analyses assessed whether associations between BMI change and hospital LOS and hospital birthing costs in the second birth differed according to GDM status across consecutive pregnancies. All analyses were performed using the statistical software Stata/SE 15. This study was reported according to the Strengthening the Reporting of Observational Studies in Epidemiology (STROBE) statement for cohort studies [32].

## 3 Results

### 3.1 Participant Characteristics

The inception cohort identified 16 111 births. Of these, 4954 women were excluded because of no second birth [*n* = 4943 women] or missing DRG/hospital LOS‐related information [*n* = 11 women]. A total of 11 157 pregnant women were included in the analysis (Table S1).

In absolute terms, 5% of women (*n* = 625/11157) had GDM in the first (index) pregnancy but not in the subsequent (second); 9% (*n* = 1012/11157) had GDM in the subsequent (second) pregnancy but not the first; and 8% (*n* = 856/11157) had GDM in both pregnancies (Tables 1 and 2). Accordingly, 13% (*n* = 1481/11157) had GDM in the first pregnancy or both, and 17% (*n* = 1868/11157) had GDM in the subsequent pregnancy or both. In relative terms, the risk of GDM in the second pregnancy was 57.8% if GDM was present in the first pregnancy (i.e., 856 with GDM in both pregnancies / 856 + 625 with GDM in the first pregnancy).

**TABLE 1:** Association between gestational diabetes mellitus diagnosis and *hospital length of stay* in the second birth.

Cohort GDM groups	*n* (%)	Second birth	Mean difference versus GDM 1	*p*
		Mean LOS days, (SD)	(95% CI)^a^	
GDM 1: No GDM diagnosed in either pregnancy (reference group)	8664 (78)	2.79 (2.12)	(reference group)	—
GDM 2: GDM in the first pregnancy but not in second pregnancy	625 (5)	3.14 (1.89)	0.35 (0.15 to 0.54)	< 0.001
GDM 3: GDM in the second pregnancy but not in first pregnancy	1012 (9)	3.43 (3.19)	0.64 (0.47 to 0.81)	< 0.001
GDM 4: GDM in both pregnancies	856 (8)	3.71 (2.92)	0.91 (0.72 to 1.11)	< 0.001
Total	11 157 (100)	2.94 (2.31)^b^		

Abbreviations: CI, confidence interval, reference category (considered as a low risk group); GDM, gestational diabetes mellitus; SD, standard deviation.

^a^ A univariate generalised linear model to assess precision estimates of mean differences.

^b^ Mean length of stay (LOS) in days, per pregnant woman.

**TABLE 2:** Association between gestational diabetes mellitus diagnosis and hospital costs (AU$) in the second birth.

Cohort GDM groups	*n* (%)	Second birth	Mean difference versus GDM 1	*p*
		Mean costs AU$, (SD)	(95% CI)^a^	
GDM 1: No GDM diagnosed in either pregnancy (reference group)	8664 (78)	$12 720 (4229)	(reference group)	—
GDM 2: GDM in the first pregnancy but not in second pregnancy	625 (5)	$13 121 (4530)	$401 ($49 to $755)	0.026
GDM 3: GDM in the second pregnancy but not in first pregnancy	1012 (9)	$14 112 (4346)	$1392 ($1090 to $1694)	< 0.001
GDM 4: GDM in both pregnancies	856 (8)	$14 680 (5062)	$1960 ($1622 to $2299)	< 0.001
Total	11 157 (100)	$13 019 (4370)^b^		

Abbreviations: CI, confidence interval, reference category (considered as a low risk group); GDM, gestational diabetes mellitus; SD, standard deviation.

^a^ A univariate generalised linear model to assess precision estimates of mean differences.

^b^ Mean costs AU$ per pregnant woman.

BMI data were missing for 14% (*n* = 1506) of women in the first pregnancy and 10% (*n* = 1078) in the second. Consequently, BMI change between pregnancies was unavailable for 15% (*n* = 1727) of the cohort (Table S1). Among women with available BMI change data, 1160 (12%) of women had a weight reduction by at least 1 BMI unit between consecutive pregnancies, 4405 (⁓47%) were stable in weight (change in BMI between −1 and < 1), and 3865 (41%) increased their weight by at least 1 BMI unit (Table 3). 59% of pregnant women experienced normal vaginal birth in the first birth (index), whereas 67% experienced the same in the second birth. 94% of births occurred after 37 completed weeks for both consecutive pregnancies (Table S1).

**TABLE 3:** Association between body mass index change and hospital length of stay in the second birth.

BMI change category between pregnancies	*n* (%)	Second birth	Mean difference versus ‘weight loss’ [group‐a]	*p*	Mean difference versus ‘stable weight’ [group‐b]	*p*
		Mean LOS days, (SD)	(95% CI)^a^		(95% CI)^a^	
Group a weight loss: (< −1 kg/m^2^)	1160 (12)	2.89 (2.14)	(reference group)	—	0.15 (0.01 to 0.29)	0.036
Group b stable weight: (−1 to < 1 kg/m^2^)	4405 (47)	2.74 (2.00)	−0.15 (−0.29 to −0.01)	0.036	(reference group)	—
Group c weight gain: (1 to < 2 kg/m^2^)	1624 (17)	2.92 (2.57)	0.03 (−0.13 to 0.20)	0.686	0.19 (0.06 to 0.31)	0.004
Group d weight gain: (2 to < 4 kg/m^2^)	1512 (16)	3.01 (1.89)	0.12 (−0.05 to 0.29)	0.160	0.27 (0.14 to 0.40)	< 0.001
Group e weight gain: (4+ kg/m^2^)	729 (8)	3.37 (3.15)	0.48 (0.25 to 0.70)	< 0.001	0.63 (0.43 to 0.83)	< 0.001
Total	9430 (100)	2.88 (2.22)^b^				

*Note:* BMI was defined as weight in kilograms divided by height in square metres (kg/m^2^) and calculated using self‐reported pre‐pregnancy weight and height recorded at the first antenatal visit. Change in BMI was calculated as the pre‐pregnancy BMI for the second birth minus the pre‐pregnancy BMI in the index birth and grouped into five categories: < −1 kg/m^2^, −1 to < 1 kg/m^2^, 1 to < 2 kg/m^2^, 2 to < 4 kg/m^2^ and 4+ kg/m^2^ (Cnattingius and Villamor [19]). Reference groups considered as (i) weight loss (Group a: (< −1 kg/m^2^)) and (ii) stable weight (Group b: (−1 to < 1 kg/m^2^)).

Abbreviations: CI, confidence interval; SD, standard deviation.

^a^ A univariate generalised linear model to assess precision estimates of mean differences.

^b^ Mean length of stay (LOS) in days, per pregnant woman.

The proportion of infants admitted to special care or neonatal intensive care was higher among women diagnosed with GDM or who gained ≥ 4 BMI units during pregnancy, compared with women with no GDM exposure and < 4‐unit BMI gain (Figure S2).

### 3.2 Hospital LOS and Costs

Table 1 shows the distribution of hospital LOS for maternal birthing costs for two consecutive pregnancies, with and without a diagnosis of GDM or change in BMI category. Among women diagnosed with GDM in both pregnancies, average LOS was 3.71 days, 0.91 days longer than for those without GDM in either pregnancy (2.79 days) for the second birth, compared with women without GDM exposure. Maternal birthing costs were higher among women diagnosed with GDM in both pregnancies. Specifically, the mean cost for the second birth was AU$14680 for these women, which was 15.41% higher (AU$1960) than the AU$12720 observed in women without GDM. In addition, compared with women with a stable BMI between pregnancies, those who gained at ≥ 4 BMI units incurred significantly higher 0.63 days (0.43–0.83) in hospital (Table 3) and birthing costs—an increase of 9% (AU$1114; 759–1470) for the second birth (Table 4).

**TABLE 4:** Association between body mass index change and hospital *costs (AU$)* in the second birth.

BMI change category between pregnancies	*n* (%)	Second birth	^a^Mean difference versus ‘weight loss’ [group‐a]	*p*	^a^Mean difference versus ‘stable weight*’* [group‐b]	*p*
		Mean costs (AU$), (SD)	(95% CI)		(95% CI)	
Group a weight loss: (< −1 kg/m^2^)	1160 (12)	$13 025 (4544)	(Reference group)	—	$370 ($92 to $649)	0.009
Group b stable weight: (−1 to < 1 kg/m^2^)	4405 (47)	$12 655 (4194)	−$370 (−$649 to −$92)	0.009	(Reference group)	—
Group c weight gain: (1 to < 2 kg/m^2^)	1624 (17)	$12 888 (4180)	‐$137 (−$463 to $188)	0.408	$233 (−$10 to $476)	*0.060*
Group d weight gain: (2 to < 4 kg/m^2^)	1512 (16)	$13 529 (4426)	$504 ($166 to $841)	0.003	$874 ($615 to $1133)	*< 0.001*
Group e weight gain: (4+ kg/m^2^)	729 (8)	$13 770 (4743)	$744 ($328 to $1160)	< 0.001	$1114 ($759 to $1470)	*< 0.001*
Total	9430 (100)	$12 967 (4334)^b^				

*Note:* BMI was defined as weight in kilograms divided by height in square metres (kg/m^2^) and calculated using self‐reported pre‐pregnancy weight and height recorded at the first antenatal visit. Change in BMI was calculated as the pre‐pregnancy BMI for the second birth minus the pre‐pregnancy BMI in the index birth and grouped into five categories: < −1 kg/m^2^, −1 to < 1 kg/m^2^, 1 to < 2 kg/m^2^, 2 to < 4 kg/m^2^ and 4+ kg/m^2^ (Cnattingius and Villamor [19]). Reference groups considered as (i) weight loss Group a: (< −1 kg/m^2^) and (ii) stable weight Group b: (−1 to < 1 kg/m^2^).

Abbreviations: CI, confidence interval; SD, standard deviation.

^a^ A univariate generalised linear model to assess precision estimates of mean differences.

^b^ Mean costs AU$ per pregnant woman.

### 3.3 Multivariable Analyses

Tables 5 and 6 present regression outputs from two separate models LOS and hospitalisation birthing costs for the second birth. The results indicate women with GDM experienced an estimated increase in LOS of 21% (16%–26%, *p* < 0.001) for the second birth compared with mothers without a GDM diagnosis in either pregnancy. In addition, maternal LOS was 9% (4%–14%, *p* < 0.001) higher among women who had a ≥ 4 BMI units weight gain than for women who achieved a stable weight (−1 to < 1 kg/m^2^) (Table 5).

**TABLE 5:** Univariate and multivariate generalised linear models assessing associations between gestational diabetes mellitus status and body mass index changes on hospital length of stay in the second birth.

Variables	Model for second birth^d^				
	Unadjusted model^b^		Adjusted model^c^		
	Exp (*β*) (95% CI)	*p*	Exp (*β*) (95% CI)	*p*	VIF
GDM groups					
No GDM in either pregnancy (reference group)	Reference group	—	Reference group	—	—
Diagnosed GDM in the first pregnancy but not in the second pregnancy	1.12 (1.07 to 1.18)	< 0.001	1.08 (1.04 to 1.13)	< 0.001	1.09
Diagnosed GDM in the second pregnancy but not in the first pregnancy	1.23 (1.16 to 1.30)	< 0.001	1.15 (1.11 to 1.20)	< 0.001	1.14
Diagnosed GDM in both pregnancies	1.33 (1.26 to 1.40)	< 0.001	1.21 (1.16 to 1.26)	< 0.001	1.14
BMI change quintile					
Group b stable weight: (−1 to < 1 kg/m^2^) (reference group)	Reference group	—	Reference group	—	—
Group a weight loss: (< −1 kg/m^2^)	1.06 (1.01 to 1.11)	0.028	1.04 (0.99 to 1.08)	0.123	1.23
Group c weight gain: (1 to < 2 kg/m^2^)	1.07 (1.02 to 1.12)	0.007	1.02 (0.99 to 1.05)	0.193	1.34
Group d weight gain: (2 to < 4 kg/m^2^)	1.10 (1.06 to 1.14)	< 0.001	1.03 (0.99 to 1.06)	0.112	1.35
Group e weight gain: (4+ kg/m^2^)	1.23 (1.15 to 1.32)	< 0.001	1.09 (1.04 to 1.14)	< 0.001	1.21
Type of delivery^a^					
Vaginal birth (reference group)	Reference group	—	Reference group	—	—
Instrumental delivery	1.24 (1.18 to 1.30)	< 0.001	1.22 (1.16 to 1.28)	< 0.001	1.06
Caesarean section‐emergency	2.10 (2.02 to 2.19)	< 0.001	1.83 (1.77 to 1.90)	< 0.001	1.21
Caesarean section‐elective	1.89 (1.82 to 1.95)	< 0.001	1.80 (1.76 to 1.85)	< 0.001	1.27
Maternal age^a^					
25–29 years (reference group)	Reference group	—	Reference group	—	—
16–24 years	1.03 (0.94 to 1.12)	0.560	1.04 (0.98 to 1.10)	0.192	1.19
30–34 years	1.05 (1.01 to 1.10)	0.026	1.02 (0.99 to 1.06)	0.257	2.20
≥ 35 years	1.17 (1.12 to 1.23)	< 0.001	1.08 (1.04 to 1.12)	< 0.001	2.25
Gestational age categories^a^					
Term (37 to 41 weeks) (reference group)	Reference group	—	Reference group	—	—
Pre‐term (< 37 weeks' gestation)	2.17 (2.01 to 2.33)	< 0.001	1.86 (1.75 to 1.98)	< 0.001	1.12
Post‐term (≥ 42 weeks)	0.96 (0.75 to 1.23)	0.745	1.00 (0.82 to 1.20)	0.960	1.00
Interpregnancy (1st birth to 2nd birth) interval in months					
< 6 months (reference group)	Reference group	—	Reference group	—	—
6–11 months	0.97 (0.91 to 1.03)	0.303	1.04 (0.98 to 1.09)	0.212	1.26
12–17 months	0.97 (0.93 to 1.02)	0.253	1.04 (0.99 to 1.08)	0.098	1.64
18–23 months	0.97 (0.93 to 1.02)	0.320	1.00 (0.96 to 1.04)	0.964	1.83
24–59 months	1.05 (1.01 to 1.10)	0.022	1.05 (1.01 to 1.08)	0.005	2.34
≥ 60 months	1.23 (1.12 to 1.35)	< 0.001	1.16 (1.06 to 1.25)	0.001	1.13
Socio‐economic disadvantage quintile					
Quintile 5 (Least disadvantaged areas) (reference group)	Reference group	—	Reference group	—	—
Quintile 1 (Most disadvantaged areas)	0.95 (0.91 to 0.99)	0.009	0.95 (0.92 to 0.99)	0.006	2.03
Quintile 2	1.02 (0.97 to 1.08)	0.425	0.99 (0.95 to 1.04)	0.742	1.34
Quintile 3	0.98 (0.94 to 1.03)	0.391	0.99 (0.95 to 1.03)	0.526	1.52
Quintile 4	0.96 (0.91 to 1.00)	0.058	0.98 (0.94 to 1.02)	0.361	1.36

*Note:* All variables were statistically significant at the 5% risk level in the crude model. Decision rules: (i) no evidence of multicollinearity problem if 0 < VIF < 5, (ii) moderate multicollinearity problem if 5 ≤ VIF ≤ 10 and (iii) serious multicollinearity problem if VIF > 10.

Abbreviations: BMI, body mass index; CI, confidence interval, reference category (considered as a low risk group); GDM, gestational diabetes mellitus; *p*‐value, the probability value; VIF, variance inflation factor.

^a^ Variable included for 2nd birth only; *β* = a regression co‐efficient using Generalised Linear Model (GLM) with a gamma family and log link function; Interpretation of exp. (*β*): a one‐unit increase in the predictor is associated with a [exp(*β*) −1] × 100% increase or decrease in the expected length of stay (LOS).

^b^ Single variable was included in the unadjusted model only.

^c^ The predictor variables were included in the adjusted model only if any label of the explanatory variable was significant at 5% or less risk level in the unadjusted model, which in turn was used to adjust for the effects of other potential confounders.

^d^ Model for second birth: ^c^Adjusted for *GDM* status, BMI change category, type of delivery, maternal age, gestational age in weeks categories, interpregnancy interval in months and socio‐economic disadvantaged quintile.

**TABLE 6:** Univariate and multivariate generalised linear models assessing associations between gestational diabetes mellitus status and body mass index change on maternal birthing hospital costs in the second birth.

Variables	Model for second birth^c^				
	Unadjusted model^d^		Adjusted model^b^		
	Exp (*β*) (95% CI)	*p*	Exp (*β*) (95% CI)	*p*	VIF
GDM groups					
No GDM in either pregnancy (reference group)	Reference group	—	Reference group	—	—
Diagnosed GDM in the first pregnancy but not in the second	1.03 (1.01 to 1.06)	0.029	1.00 (0.98 to 1.02)	0.610	1.09
Diagnosed GDM in the second pregnancy but not in the first pregnancy	1.10 (1.08 to 1.13)	< 0.001	1.07 (1.05 to 1.08)	< 0.001	1.14
Diagnosed GDM in both pregnancies	1.15 (1.12 to 1.18)	< 0.001	1.09 (1.06 to 1.11)	< 0.001	1.14
BMI change quintile					
Group b stable weight: (−1 to < 1 kg/m^2^) (reference group)	Reference group	—	Reference group	—	—
Group a weight loss: (< −1 kg/m^2^)	1.03 (1.01 to 1.05)	0.011	1.00 (0.98 to 1.02)	0.446	1.23
Group c weight gain: (1 to < 2 kg/m^2^)	1.02 (1.00 to 1.03)	0.054	0.99 (0.97 to 1.00)	0.188	1.34
Group d weight gain: (2 to < 4 kg/m^2^)	1.06 (1.04 to 1.08)	< 0.001	1.01 (0.99 to 1.02)	0.189	1.35
Group e weight gain: (4+ kg/m^2^)	1.08 (1.05 to 1.11)	< 0.001	1.01 (0.99 to 1.03)	0.264	1.21
Type of delivery^a^					
Vaginal birth (reference group)	Reference group	—	Reference group	—	—
Instrumental delivery	1.10 (1.08 to 1.13)	< 0.001	1.09 (1.06 to 1.13)	< 0.001	1.06
Caesarean section‐emergency	1.62 (1.59 to 1.65)	< 0.001	1.57 (1.54 to 1.60)	< 0.001	1.21
Caesarean section‐elective	1.53 (1.52 to 1.55)	< 0.001	1.52 (1.50 to 1.54)	< 0.001	1.27
Maternal age^a^					
25–29 years (reference group)	Reference group	—	Reference group	—	—
16–24 years	0.96 (0.93 to 1.00)	0.073	1.01 (0.98 to 1.04)	0.410	1.20
30–34 years	1.03 (1.01 to 1.05)	< 0.001	1.00 (0.98 to 1.01)	0.615	2.20
≥ 35 years	1.06 (1.04 to 1.08)	< 0.001	1.00 (0.99 to 1.02)	0.487	2.25
Gestational age categories^a^					
Term (37 to 41 weeks) (reference group)	Reference group	—	Reference group	—	—
Pre‐term (< 37 weeks' gestation)	1.27 (1.23 to 1.32)	< 0.001	1.16 (1.12 to 1.21)	< 0.001	1.12
Post‐term (≥ 42 weeks)	1.08 (0.94 to 1.24)	0.265	1.10 (1.01 to 1.20)	0.029	1.00
Interpregnancy (1st birth to 2nd birth) interval in months					
< 6 months (reference group)	Reference group	—	Reference group	—	—
6–11 months	0.89 (0.86 to 0.93)	< 0.001	0.93 (0.90 to 0.96)	< 0.001	1.26
12–17 months	0.94 (0.92 to 0.96)	< 0.001	0.96 (0.94 to 0.97)	< 0.001	1.64
18–23 months	0.98 (0.97 to 1.00)	0.113	0.99 (0.98 to 1.01)	0.775	1.83
24–59 months	1.02 (1.00 to 1.04)	0.036	1.00 (0.99 to 1.02)	0.336	2.34
≥ 60 months	1.04 (0.99 to 1.09)	0.100	0.99 (0.95 to 1.03)	0.875	1.13
Socio‐economic disadvantage quintile					
Quintile 5 (Least disadvantaged areas) (reference group)	Reference group	—	Reference group	—	—
Quintile 1 (Most disadvantaged areas)	0.99 (0.97 to 1.01)	0.492	0.98 (0.96 to 0.99)	0.002	2.03
Quintile 2	1.00 (0.98 to 1.02)	0.620	1.00 (0.98 to 1.01)	0.751	1.34
Quintile 3	1.01 (0.99 to 1.03)	0.093	1.00 (0.98 to 1.01)	0.948	1.52
Quintile 4	0.97 (0.96 to 1.00)	0.041	0.98 (0.96 to 0.99)	0.044	1.36

*Note:* *β* = a regression co‐efficient using Generalised Linear Model (GLM) with a gamma family and log link function; Interpretation of exp. (*β*): a one‐unit increase in the predictor is associated with a [exp(*β*) −1] × 100% increase or decrease in the expected maternal hospitalisation birthing costs. All variables were statistically significant at the 5% risk level in the crude model. Decision rules: (i) no evidence of multicollinearity problem if 0 < VIF < 5, (ii) moderate multicollinearity problem if 5 ≤ VIF ≤ 10 and (iii) serious multicollinearity problem if VIF > 10.

Abbreviations: BMI, body mass index; CI, confidence interval, reference category (considered as a low risk group); GDM, gestational diabetes mellitus; *p*‐value = the probability value; VIF, variance inflation factor.

^a^ Variable included for 2nd birth only.

^b^ The predictor variables were included in the adjusted model only if any label of the explanatory variable was significant at 5% or less risk level in the unadjusted model, which in turn was used to adjust for the effects of other potential confounders.

^c^ Model for second birth: Adjusted for *GDM* status, *BMI* change category, type of delivery, maternal age, gestational age in weeks categories, interpregnancy interval in months and socio‐economic disadvantaged quintile.

^d^ Single variable was included in the unadjusted model only.

Table 6 shows that women with GDM in both pregnancies had 9% higher hospital birthing costs (6%–11%, *p* < 0.001) for the second birth compared with those without a GDM diagnosis in either pregnancy. In contrast, women diagnosed with GDM only in the second pregnancy incurred 7% higher birthing costs (5%–8%, *p* < 0.001) (Table 6). Sensitivity analyses showed the robustness of these associations (Tables: S2.1, S2.2, S3.1 and S3.2).

We additionally assessed effect modification by interpregnancy BMI change by including interaction terms between longitudinal GDM status and changes in BMI category. Key interaction results are summarised in Table S4. Notably, women with GDM diagnosis and high interpregnancy weight gain (≥ 4 kg/m^2^) experienced a 23% increase in LOS (12%–36%, *p* < 0.001) and a 9% increase in hospitalisation birthing costs (4%–15%, *p* < 0.001) for the second birth, compared with the reference group (no GDM in either pregnancy and stable weight).

## 4 Discussion

### 4.1 Main Results

Our retrospective cohort study findings indicated a GDM diagnosis, or an increase in maternal BMI between pregnancies, was associated with longer hospital stays and increased maternal birthing costs during the second birth. Notably, 17% of women had a GDM diagnosis at the subsequent (second) birth, defined as the next recorded pregnancy following the index birth, and the relative risk of GDM in the second birth given GDM in the first birth was 58%. Similarly, a BMI increase of 4 or more units was associated with longer hospital length of stay and higher birthing costs in the second birth. These associations decreased only slightly when adjusted for all other factors.

Importantly, interaction analyses indicated the association between interpregnancy BMI change and both LOS and costs differed by longitudinal GDM status. In particular, women with GDM who also experienced high interpregnancy BMI gain (≥ 4 kg/m^2^) incurred the greatest increases in hospital use, including a 23% increase in LOS and a 9% increase in birthing costs in the second birth. These findings suggest GDM and excessive interpregnancy weight gain may act synergistically to increase maternal hospital utilisation. Although the absolute differences were modest (e.g., ~0.9 days longer LOS and ~9% higher birth‐admission costs), they are operationally meaningful when applied across large numbers of births at the population level. Small per‐admission increases in LOS accumulate into additional bed‐days, staffing demand and capacity pressures, particularly in higher‐risk subgroups such as for women with GDM and substantial interpregnancy BMI gain.

### 4.2 Results in the Context of What Is Known

The finding of 58% relative risk of recurrent GDM is consistent with international evidence, where reported recurrence rates typically range from 30% to 70%, dependent on population characteristics, diagnostic criteria and interpregnancy interval [33, 34, 35]. The association between GDM and higher LOS and costs for a single birth has been reported to some degree in other studies [22, 23, 25, 36, 37, 38]; however, our study extends this evidence by linking consecutive pregnancies to quantify LOS and hospital birth‐admission costs at a subsequent (second) birth according to longitudinal GDM status and interpregnancy BMI change. Focusing on the second birth captures within‐woman changes in risk between pregnancies (e.g., new, persistent, or resolved GDM and substantial interpregnancy BMI gain) and identifies subgroups with concentrated excess hospital utilisation and costs, helping us to determine when and for whom interpregnancy monitoring and targeted preventive interventions may be most beneficial.

Increased health system costs are likely to be due to a higher likelihood of adverse pregnancy outcomes (e.g., fetal malformation) [39], cardiometabolic risk factors (e.g., lipid abnormalities) [40, 41, 42, 43, 44], increased severity of cardiac disease [42], and long‐term complications [45]. In our DRG‐based costing, higher birth‐admission costs among women with GDM were largely driven by a greater share of higher‐cost (higher‐weight) maternal AR‐DRG classifications and by admissions that included neonatal hospitalisation, together with longer maternal LOS. These DRG‐based patterns support the plausibility of the observed associations between GDM and interpregnancy BMI gain and higher costs, although causal inference cannot be assumed from the observational design. Pregnant women with GDM require more advanced health care [46] and need additional medication [45], which places a considerable economic burden on midwifery, obstetric and diabetes health services for appropriate diagnosis and management.

The incidence of GDM can be reduced through exercise and dietary interventions [47, 48, 49]. The economic burden of GDM can potentially be reduced through prevention [44, 50, 51, 52, 53, 54], through Government programs such as Get Healthy, supporting weight loss prior to pregnancy and minimising weight gain, universal GDM screening and enhanced glucose control among diagnosed women [55, 56, 57, 58]. Our study supports the importance of aiming to achieve and, where feasible, maintain an optimal/normal body weight between pregnancies. Engaging in regular physical exercise, maintaining a balanced, nutritious diet and encouraging overweight and obese women to lose weight prior to the next pregnancy may reduce subsequent GDM [49, 59]. Improved overall health status, although challenging to achieve, is likely to contribute to shorter hospital LOS. Interpregnancy BMI gain is a recognised independent risk factor for GDM, but its contribution is typically modest and should be interpreted within a multifactorial risk profile [60, 61]. BMI change is not the only determinant of GDM; other factors such as maternal age, baseline BMI, prior GDM, family history of diabetes, ethnicity and socioeconomic circumstances also contribute to risk and may influence subsequent healthcare utilisation. Accordingly, prevention and monitoring strategies should consider BMI change alongside other clinical and sociodemographic risk factors rather than as a standalone predictor.

Our findings, which show a significant association between interpregnancy BMI unit increase and higher medical costs consistent with studies in other countries [12, 13, 21, 62, 63, 64]. Numerous studies have reported more adverse pregnancy outcomes associated with obesity [19, 64, 65]. These include an increased risk of instrumental or caesarean delivery [66, 67] and chronic comorbidities [68, 69]. Even for operative deliveries, total theatre, surgical and anaesthesia time are greater for women with high BMI change compared with women with no BMI change [63], leading to higher total hospital and operating theatre costs [70]. Although our study captured total hospital birthing costs, existing literature supports increased use of inpatient and outpatient services among obese women [21], especially with comorbidities [71]. Future studies could use routinely collected (administrative) data to quantify total cost of primary care and in‐patient healthcare use among women with elevated pre‐pregnancy BMI.

Our interaction findings extend this evidence by showing the adverse associations of interpregnancy BMI gain with LOS and costs are most pronounced among women with GDM across consecutive pregnancies [8]. This pattern is clinically plausible, as high BMI gain is associated with metabolic dysregulation and may amplify pregnancy complications and care intensity among women with GDM [72, 73], thereby increasing hospital utilisation and costs.

### 4.3 Implications for Policy and Clinical Practice

Our study provides evidence of the economic impact to health systems of a change in GDM status and BMI increase across consecutive pregnancies. Policy and practice initiatives that promote healthy weight between pregnancies may help reduce the burden associated with GDM and interpregnancy BMI gain; however, our observational study cannot determine whether these interventions would reduce LOS or costs. Importantly, the ≥ 4 kg/m^2^ interpregnancy BMI gain associated with longer LOS represents a substantial weight increase, approximately 10–13 kg for women of typical heights (e.g., 1.60–1.80 m; BMI change × height [2]), highlighting a clinically meaningful and potentially modifiable risk marker. These initiatives should emphasise and coordinate a collaborative approach across primary and specialist care, including allied health professionals. Evidence from recent meta‐analysis suggested group‐based physical activity interventions can significantly reduce the risk of GDM by 34% [48]. This underscores the need for comprehensive preventive strategies that combine weight management with accessible, community‐based activity programs. Examples of public health and service‐level approaches that have been proposed or evaluated elsewhere include subsidies for healthy foods, taxes on sugary drinks, nutrition education for reproductive‐age women, community wellness programs and enhanced maternity care with dietitian support. These examples are provided for context and should be interpreted as illustrative rather than evidenced from the present study. Such an approach aligns with the ‘Australian National Diabetes Strategy 2021–2030’, goal‐four [74], which prioritises improving maternal health and pregnancy outcomes for reproductive women in Australia and worldwide. Interpregnancy interval may also influence these patterns: shorter intervals may limit time to return to pre‐pregnancy healthy weight (postpartum weight retention), whereas longer intervals may observe gradual weight gain with increasing age and changing lifestyle factors. These explanations are somewhat speculative, but together suggest that interpregnancy weight‐management support may be important for both short and longer intervals. Furthermore, pregnant women who experience high‐level interpregnancy weight gain should be carefully monitored during their second pregnancy to identify GDM, irrespective of pre‐pregnancy BMI. Antenatal guidelines for observing GDM in pregnancy should include interpregnancy weight change as an independent risk factor for GDM with a glucose tolerance test in women with high weight gain. However, interpregnancy BMI change should be considered as one component of risk rather than a standalone predictor, and monitoring and prevention efforts should be guided by a multifactorial risk assessment. A possible preventive effect on GDM incidence of losing weight between pregnancies among women with overweight/obesity needs to be replicated in other studies. Public health efforts that target overweight women in pregnancy and childbirth could expand in focus to promote healthy weight from pre‐conception throughout reproduction [75, 76]. Today, fewer than 10% of countries' national policies address healthy maternal weight across the entire spectrum of childbearing [77]. Future interventional and implementation studies are needed to determine whether interpregnancy weight‐management strategies translate into observed reductions in GDM recurrence, LOS and hospital costs, and to quantify subsequent cost savings.

### 4.4 Strengths and Limitations

The main strength of this study was the use of a large cohort of consecutive births in two hospitals. Using electronic medical record data, we constructed a cohort of women with two most recent singleton births: the index birth (regardless of GDM) and the next birth, linked via unique national identifiers. Consistent exposure/outcome definitions over time ensured internal consistency. The long accrual period and large sample provided precise estimates, and we adjusted for multiple known confounders to isolate the contributions of BMI change and GDM to LOS and costs. These features support validity, reliability and generalisability to managers, policymakers, obstetric clinicians, midwives and pregnant women in Australia and similar health systems.

This study has some limitations. First, as an observational cohort study, the findings are associative and do not establish causation. Although we adjusted for measured covariates, residual confounding and unmeasured factors (e.g., diet quality, physical activity, smoking intensity, clinical management differences and severity of hyperglycaemia) may remain and could partly explain observed differences in LOS and costs. Our primary analyses were conducted using a complete‐case analysis approach, and exclusion due to missing outcome information (LOS and/or AR‐DRG–derived cost data) may introduce selection bias if missingness differed by exposure status. We did not undertake additional analyses to formally assess the impact of this potential bias. Second, the diagnostic criteria for GDM changed during the study period (shifting from the pre‐2015 two‐step approach to the post‐2015 ADIPS one‐step 75‐g OGTT with a lower fasting threshold). Because we identified GDM using ICD‐10‐AM O24.4 at birth admission rather than laboratory results, we could not harmonise case definitions across diagnostic eras. This results in some case‐definition heterogeneity due to temporal differences in ascertainment. Any resulting bias is unlikely to be differential with respect to length of stay and cost outcomes and would most likely attenuate observed associations. Nonetheless, residual bias cannot be ruled out, and findings should be interpreted with appropriate caution. Third, BMI was missing for a proportion of women; primary analyses used a complete case approach, with sensitivity analyses using imputed BMI change values (from bootstrapped models) showing broadly consistent results, although some uncertainty remains. Fourth, the analysis was confined to assessment of hospital maternal birthing costs and did not capture broader healthcare use (e.g., outpatient visits, allied health, pharmaceuticals), indirect costs (e.g., time off work, productivity loss), or longer‐term maternal and child outcomes. Pregnant women with GDM/BMI increases may incur greater indirect costs–such as increased time off from work, psychological stress, or reduced productivity in daily activities, than women without GDM/BMI increases. Long‐term maternal/child outcomes, especially progression to type 2 diabetes [78]. As a result, total costs attributable to GDM and interpregnancy BMI gain may be underestimated. Pre‐pregnancy height/weight were self‐reported, risking under‐reporting, though a recent review indicates such measures are valid and do not bias associations between interpregnancy weight change and birth outcomes [79]. Fifth, the second‐birth period (2011–2019) spans potential changes in perinatal care pathways or administrative coding (including updates to ICD‐10‐AM/AR‐DRG and evolving GDM management and admission/discharge practices). Although AR‐DRG–based costs were applied consistently and expressed in a common reference year, residual temporal confounding cannot be excluded. Sixth, findings are generalisable to similar Australian hospital settings; while the direction of associations is likely relevant to current practice, absolute LOS and cost estimates may differ under contemporary and future maternity care models and pricing/coding arrangements.

## 5 Conclusion

Our study suggests that subsequent‐pregnancy GDM (new or persistent) and interpregnancy BMI increases are associated with higher maternal hospital LOS and birthing costs. These findings underscore the need for prevention programs and public health policies to lessen GDM and excessive interpregnancy weight gain, lower healthcare expenditure, and improve maternal and long‐term offspring health. Effective preventive approaches may include weight‐management interventions, such as nutritional counselling, structured lifestyle modification, physical activity and a healthy diet, which could be implemented in primary care and community settings to support reproductive women planning a second pregnancy. Further studies should evaluate the cost‐effectiveness of programs targeting women at risk of GDM or overweight/obesity.

## Author Contributions

**Rashidul Alam Mahumud:** conceptualisation, investigation, methodology, data curation, formal analysis, writing – original draft, writing – review and editing. **Glynis Pauline Ross:** investigation, methodology, writing – review and editing. **Adam Mackie:** investigation, methodology, writing – review and editing. **Rachael L. Morton:** conceptualisation, investigation, methodology, data curation, writing – original draft, writing – review and editing, supervision. **Kirsten I. Black:** conceptualisation, investigation, ethical approvals, methodology, data curation, writing – original draft, writing – review and editing.

## Funding

Rachael Morton is supported by a NHMRC Investigator award #1194703. Mid‐career Researcher Accelerator Grant from the Faculty of Medicine and Health, University of Sydney to Kirsten Black to undertake the study.

## Ethics Statement

Ethical approval for this study was obtained from the Sydney Local Health District Research Ethics Committee (Royal Prince Alfred Hospital [RPAH] Zone), a Human Research Ethics Committee (HREC). The committee approved the analysis of archived electronic health records under HREC reference number 2019/ETH06421, with approval granted on 23 March 2019.

## Consent

The authors have nothing to report.

## Conflicts of Interest

The authors declare no conflicts of interest.

## Supporting information


**Figure S1.** Distribution of hospitalisation length of stay and maternal medical birthing costs.
**Figure S2.** Distribution of infant special care or neonatal intensive care by GDM status and BMI change category.

**Table S1.** Maternal background characteristics during pregnancy.
**Table S2.1.** Sensitivity analysis for hospital length of stay in the second birth, using a multivariate generalised linear model with imputed values for missing BMI change.
**Table S2.2.** Bootstrapped sensitivity analysis for hospital length of stay in the second birth, using a multivariate generalised linear model with imputed values for missing BMI change.
**Table S3.1.** Sensitivity analysis for maternal hospitalisation costs in the second birth, using a multivariate generalised linear model with imputed values for missing BMI change.
**Table S3.2.** Bootstrapped sensitivity analysis for maternal hospital birthing costs in the second birth, using a multivariate generalised linear model with imputed values for missing BMI change.
**Table S4.** Interaction effect of gestational diabetes mellitus and body mass index change on maternal hospital length of stay and medical birthing costs in the second birth.

## Data Availability

The data that support the findings of this study are available on request from the corresponding author. The data are not publicly available due to privacy or ethical restrictions.
