# Supplementary material for: The Economic Burden of Gestational Diabetes and Body Mass Index Changes Between Pregnancies: A Retrospective Cohort Study
Source: BJOG. 2026 Mar 10;133(8):1602–15. doi: 10.1111/1471-0528.70208 (PMC13254038; doi:10.1111/1471-0528.70208)

**Supplementary Figures:**

Figure S1. Distribution of hospitalisation length of stay and maternal medical birthing costs in the second birth.

Figure S2. Distribution of infant special care or neonatal intensive care for by GDM status and BMI change category


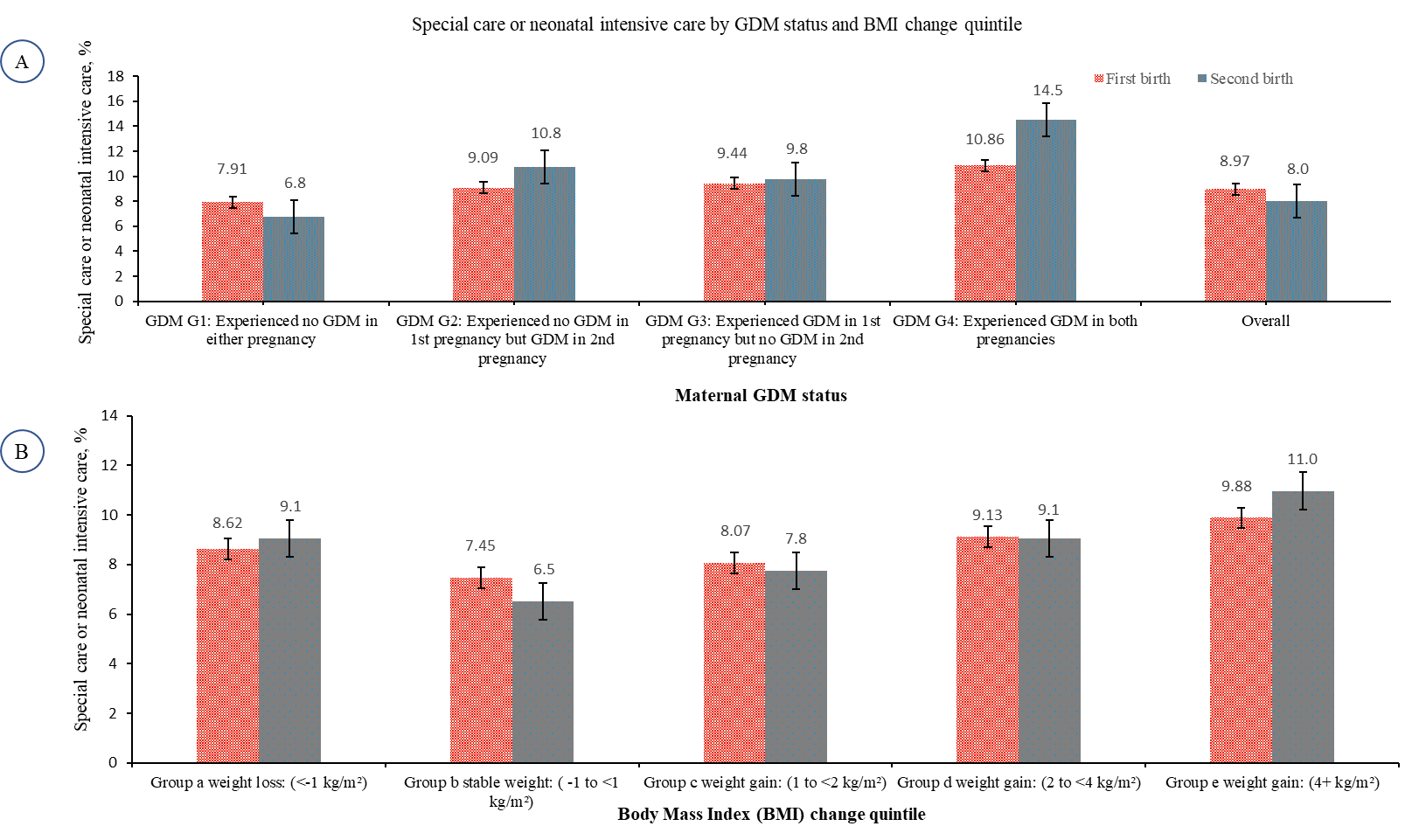

Supplement: Supplementary file 1 — Figure S1. Distribution of hospitalisation length of stay and maternal medical birthing costs. Figure S2. Distribution of infant special care or neonatal intensive care by GDM status and BMI change category. [file BJO-133-1602-s002.docx]
