# Supplementary material for: The Economic Burden of Gestational Diabetes and Body Mass Index Changes Between Pregnancies: A Retrospective Cohort Study
Source: BJOG. 2026 Mar 10;133(8):1602–15. doi: 10.1111/1471-0528.70208 (PMC13254038; doi:10.1111/1471-0528.70208)
Supplement: Supplementary file 2 — Table S1. Maternal background characteristics during pregnancy. Table S2.1. Sensitivity analysis for hospital length of stay in the second birth, using a multivariate generalised linear model with imputed values for missing BMI change. Table S2.2. Bootstrapped sensitivity analysis for hospital length of stay in the second birth, using a multivariate generalised linear model with imputed values for missing BMI change. Table S3.1. Sensitivity analysis for maternal hospitalisation costs in the second birth, using a multivariate generalised linear model with imputed values for missing BMI change. Table S3.2. Bootstrapped sensitivity analysis for maternal hospital birthing costs in the second birth, using a multivariate generalised linear model with imputed values for missing BMI change. Table S4. Interaction effect of gestational diabetes mellitus and body mass index change on maternal hospital length of stay and medical birthing costs in the second birth. [file BJO-133-1602-s001.docx]

**Supplementary Tables**

Table S1. Maternal background characteristics during pregnancy

| Variables | First birth | | Second birth | |  |
| --- | --- | --- | --- | --- | --- |
|  | **n** | **% / Mean (SD)** | **n** | **% / Mean (SD)** |  |
| GDM groups, n (%) |  |  |  |  |  |
| *No diagnosed GDM in either pregnancy* | 8,664 (78) | | | | |
| *Diagnosed GDM in 1st pregnancy but no GDM in 2nd pregnancy* | 625 (5) | | | | |
| *Diagnosed no GDM in 1st pregnancy but GDM in 2nd pregnancy* | 1,012 (9) | | | | |
| *Diagnosed GDM in both pregnancies* | 856 (8) | | | | |
| BMI change category (first pregnancy to 2nd pregnancy , n (%) |  |  |  |  |  |
| *Group a weight loss: (<-1 kg/m²)* | 1160 (10) | | | | |
| *Group b stable weight: ( -1 to <1 kg/m²)* | 4405 (39) | | | | |
| *Group c weight gain: (1 to <2 kg/m²)* | 1624 (15) | | | | |
| *Group d weight gain: (2 to <4 kg/m²)* | 1512 (14) | | | | |
| *Group e weight gain: (*≥*4 kg/m²)* | 729 (7) | | | | |
| *Missing observation* | 1727 (15) | | | | |
| Type of delivery, n (%) |  |  |  |  |  |
| *Normal vaginal birth* | 6,612 | 59.26 | 7,525 | 67.45 |  |
| *Instrumental delivery* | 1,793 | 16.07 | 452 | 4.05 |  |
| *Caesarean section-emergency* | 1,692 | 15.17 | 1,072 | 9.61 |  |
| *Caesarean section-elective* | 1,054 | 9.45 | 2,083 | 18.67 |  |
| *Missing observation* | 6 | 0.05 | 25 | 0.22 |  |
| Maternal hospitalisation length of stay (LOS), mean (SD) | 11,154 | 3.63 days (2.21) | 11,141 | 2.94 days (2.31) |  |
| Maternal birthing costs, mean (SD) | 11157 | AU$ 13,182 (4,182) | 11157 | AU$ 13,019 (4,370) |  |
| Birth year, n (%) |  |  |  |  |  |
| ≤ *2011* | 2,029 | 18.19 | 9 | 0.08 |  |
| *2012-2013* | 3,743 | 33.55 | 1,123 | 10.07 |  |
| *2014-2015* | 3,539 | 31.72 | 2,909 | 26.07 |  |
| *2016-2017* | 1,827 | 16.38 | 4,178 | 37.45 |  |
| *2018-2019* | 16 | 0.14 | 2,932 | 26.28 |  |
| *Missing observation* | 3 | 0.03 | 6 | 0.05 |  |
| Maternal age in year, n (%) |  |  |  |  |  |
| *16-24 years* | 1,212 | 10.86 | 483 | 4.33 |  |
| *25-29 years* | 3,144 | 28.18 | 1,994 | 17.87 |  |
| *30-34 years* | 4,511 | 40.43 | 4,175 | 37.42 |  |
| ≥ *35 years* | 2,290 | 20.53 | 4,505 | 40.38 |  |
| Gestational age in weeks, mean (SD) | 11,157 | 38.85 (2.30) | 11,157 | 38.64 (2.03) |  |
| Gestational age in weeks, n (%) |  |  |  |  |  |
| Pre-term (<37 weeks' gestation) | 692 | 6.20 | 698 | 6.26 |  |
| Term (37 to 41 weeks) | 10,424 | 93.43 | 10,439 | 93.56 |  |
| Post-term (≥ 42 weeks) | 41 | 0.37 | 20 | 0.18 |  |
| Average years of pregnancy interval between first and second birth, mean (SD) | 1.83 (1.15) | | | |  |
| Interpregnancy (first birth to second birth) interval in months, n (%) |  |  |  |  |  |
| *<6 months* | 1,952 (17.50) | | | | |
| *6-11 months* | 668 (5.99) | | | | |
| *12-17 months* | 2,007 (17.99) | | | | |
| *18-23 months* | 2,657 (23.81) | | | | |
| *24-59 months* | 3,610 (32.36) | | | | |
| *≥ 60 months* | 263 (2.36) | | | | |
| *Socio-economic disadvantage quintile, n (%)* |  | | | | |
| *Quintile 1 (Most disadvantaged areas)* | 3,182 (28.52) | | | | |
| *Quintile 2* | 1,272 (11.40) | | | | |
| *Quintile 3* | 1,981 (17.76) | | | | |
| *Quintile 4* | 1,408 (12.62) | | | | |
| *Quintile 1 (Least disadvantaged areas)* | 3,143 (28.17) | | | | |
| *Missing observation* | 171 (1.53) | | | | |

SD = standard deviation

Table S2.1 Sensitivity analysis for hospital length of stay in the second birth, using a multivariate generalised linear model with imputed values for missing BMI change

| Variables | Adjusted model^€^ | |
| --- | --- | --- |
|  | Exp (β) (95% CI) | p-value |
| GDM groups |  |  |
| No GDM in either pregnancy (reference group) | reference group | - |
| Diagnosed GDM in the first pregnancy but not in the second | 1.04 (1.01 to 1.08) | 0.009 |
| Diagnosed GDM in the second pregnancy but not in the first pregnancy | 1.15 (1.13 to 1.18) | <0.001 |
| Diagnosed GDM in both pregnancies | 1.17 (1.14 to 1.20) | <0.001 |
| BMI change quintile |  |  |
| Group b stable weight: (-1 to <1 kg/m²) (reference group) | reference group | - |
| Group a weight loss: (<-1 kg/m²) | 1.05 (1.03 to 1.08) | <0.001 |
| Group c weight gain: (1 to <2 kg/m²) | 1.01 (0.98 to 1.04) | 0.412 |
| Group d weight gain: (2 to <4 kg/m²) | 1.02 (0.99 to 1.04) | 0.143 |
| Group e weight gain: (≥4 kg/m²) | 1.08 (1.06 to 1.10) | <0.001 |
| Type of delivery^§^ |  |  |
| Vaginal birth (reference group) | reference group | - |
| Instrumental delivery | 1.23 (1.19 to 1.27) | <0.001 |
| Caesarean section-emergency | 1.94 (1.90 to 1.99) | <0.001 |
| Caesarean section-elective | 1.89 (1.86 to 1.92) | <0.001 |
| Maternal age^§^ |  |  |
| 25-29 years (reference group) | reference group | - |
| 16-24 years | 1.21 (1.14 to 1.29) | <0.001 |
| 30-34 years | 1.02 (0.99 to 1.05) | 0.133 |
| ≥ 35 years | 1.05 (1.02 to 1.08) | 0.001 |
| Gestational age categories^§^ |  |  |
| Term (37 to 41 weeks) (reference group) | reference group | - |
| Pre-term (<37 weeks' gestation) | 1.76 (1.68 to 1.84) | <0.001 |
| Post-term (≥ 42 weeks) | 0.95 (0.79 to 1.14) | 0.549 |
| Interpregnancy (1^st^ birth to 2^nd^ birth) interval in months |  |  |
| <6 months (reference group) | reference group | - |
| 6-11 months | 0.98 (0.94 to 1.01) | 0.201 |
| 12-17 months | 1.01 (0.98 to 1.03) | 0.531 |
| 18-23 months | 1.04 (1.02 to 1.06) | 0.001 |
| 24-59 months | 1.06 (1.04 to 1.08) | <0.001 |
| ≥ 60 months | 1.24 (1.16 to 1.32) | <0.001 |
| Socio-economic disadvantage quintile |  |  |
| Quintile 5 (Least disadvantaged areas) (reference group) | reference group | - |
| Quintile 1 (Most disadvantaged areas) | 1.02 (0.99 to 1.04) | 0.146 |
| Quintile 2 | 1.10 (1.07 to 1.13) | <0.001 |
| Quintile 3 | 1.01 (0.99 to 1.03) | 0.297 |
| Quintile 4 | 1.01 (0.99 to 1.03) | 0.380 |

Note: ^§^Variable included for 2^nd^ birth only; β = a regression co-efficient using Generalised Linear Model (*GLM*) with a gamma family and log link function; Interpretation of exp (β): a one-unit increase in the predictor is associated with a [exp(β) −1]×100% increase or decrease in the expected length of stay (LOS); *CI* = confidence interval, reference category (considered as a low risk group); [*GDM* = gestational diabetes mellitus;](https://www.hopkinsmedicine.org/health/conditions-and-diseases/diabetes/gestational-diabetes) BMI = body mass index; p-value = the probability value; all variables were statistical significance at the 5% risk level in the crude model; ^€^The predictor variables were included in the adjusted model only if any label of the explanatory variable was significant at 5% or less risk level in the unadjusted model, which in turn was used to adjust for the effects of other potential confounders; ^€^Model for second birth: Adjusted for *GDM* status, *BMI* change category, type of delivery, maternal age, gestational age in weeks categories, interpregnancy interval in months and socio-economic disadvantaged quintile.

Table S2.2 Bootstrapped sensitivity analysis for hospital length of stay in the second birth, using a multivariate generalised linear model with imputed values for missing BMI change

| Variables | Adjusted model for second birth^€^ | |
| --- | --- | --- |
|  | Exp (β) (95% CI) | p-value |
| GDM groups |  |  |
| No GDM in either pregnancy (reference group) | reference group | - |
| Diagnosed GDM in the first pregnancy but not in the second | 1.08 (1.04 to 1.13) | <0.001 |
| Diagnosed GDM in the second pregnancy but not in the first pregnancy | 1.15 (1.11 to 1.20) | <0.001 |
| Diagnosed GDM in both pregnancies | 1.21 (1.16 to 1.26) | <0.001 |
| BMI change quintile |  |  |
| Group b stable weight: ( -1 to <1 kg/m²) (reference group) | reference group | - |
| Group a weight loss: (<-1 kg/m²) | 1.04 (0.99 to 1.08) | 0.119 |
| Group c weight gain: (1 to <2 kg/m²) | 1.02 (0.99 to 1.05) | 0.192 |
| Group d weight gain: (2 to <4 kg/m²) | 1.03 (0.99 to 1.06) | 0.116 |
| Group e weight gain: (≥4 kg/m²) | 1.09 (1.04 to 1.14) | <0.001 |
| Type of delivery^§^ |  |  |
| Vaginal birth (reference group) | reference group | - |
| Instrumental delivery | 1.22 (1.16 to 1.28) | <0.001 |
| Caesarean section-emergency | 1.83 (1.77 to 1.90) | <0.001 |
| Caesarean section-elective | 1.80 (1.76 to 1.85) | <0.001 |
| Maternal age^§^ |  |  |
| 25-29 years (reference group) | reference group | - |
| 16-24 years | 1.04 (0.98 to 1.10) | 0.194 |
| 30-34 years | 1.02 (0.99 to 1.06) | 0.255 |
| ≥ 35 years | 1.08 (1.04 to 1.12) | <0.001 |
| Gestational age categories^§^ |  |  |
| Term (37 to 41 weeks) (reference group) | reference group | - |
| Pre-term (<37 weeks' gestation) | 1.86 (1.75 to 1.98) | <0.001 |
| Post-term (≥ 42 weeks) | 1.00 (0.81 to 1.22) | 0.962 |
| Interpregnancy (1^st^ birth to 2^nd^ birth) interval in months |  |  |
| <6 months (reference group) | reference group | - |
| 6-11 months | 1.04 (0.98 to 1.09) | 0.204 |
| 12-17 months | 1.04 (0.99 to 1.08) | 0.098 |
| 18-23 months | 1.00 (0.96 to 1.04) | 0.963 |
| 24-59 months | 1.05 (1.01 to 1.08) | 0.006 |
| ≥ 60 months | 1.16 (1.06 to 1.26) | 0.001 |
| Socio-economic disadvantage quintile |  |  |
| Quintile 5 (Least disadvantaged areas) (reference group) | reference group | - |
| Quintile 1 (Most disadvantaged areas) | 0.95 (0.92 to 0.99) | 0.006 |
| Quintile 2 | 0.99 (0.95 to 1.04) | 0.743 |
| Quintile 3 | 0.99 (0.95 to 1.03) | 0.525 |
| Quintile 4 | 0.98 (0.94 to 1.02) | 0.357 |

Note: ^§^Variable included for 2^nd^ birth only; β = a regression co-efficient using Generalised Linear Model (*GLM*) with a gamma family and log link function using 10,000 replications; Interpretation of exp (β): a one-unit increase in the predictor is associated with a [exp(β) −1]×100% increase or decrease in the expected length of stay (LOS); *CI* = confidence interval, reference category (considered as a low risk group); [*GDM* = gestational diabetes mellitus;](https://www.hopkinsmedicine.org/health/conditions-and-diseases/diabetes/gestational-diabetes) BMI = body mass index; p-value = the probability value; all variables were statistical significance at the 5% risk level in the crude model; ^€^The predictor variables were included in the adjusted model only if any label of the explanatory variable was significant at 5% or less risk level in the unadjusted model, which in turn was used to adjust for the effects of other potential confounders; ^€^Model for second birth: Adjusted for *GDM* status, *BMI* change category, type of delivery, maternal age, gestational age in weeks categories, interpregnancy interval in months and socio-economic disadvantaged quintile.

Table S3.1 Sensitivity analysis for maternal hospital costs in the second birth, using a multivariate generalised linear model with imputed values for missing BMI change

| Variables | Adjusted model for second birth^€^ | |
| --- | --- | --- |
|  | Exp (β) (95% CI) | p-value |
| GDM groups |  |  |
| No GDM in either pregnancy (reference group) | reference group | - |
| Diagnosed GDM in the first pregnancy but not in the second | 1.00 (0.98 to 1.03) | 0.605 |
| Diagnosed GDM in the second pregnancy but not in the first pregnancy | 1.07 (1.05 to 1.09) | <0.001 |
| Diagnosed GDM in both pregnancies | 1.09 (1.07 to 1.11) | <0.001 |
| BMI change quintile |  |  |
| Group b stable weight: ( -1 to <1 kg/m²) (reference group) | reference group | - |
| Group a weight loss: (<-1 kg/m²) | 1.01 (0.99 to 1.02) | 0.424 |
| Group c weight gain: (1 to <2 kg/m²) | 0.99 (0.97 to 1.00) | 0.205 |
| Group d weight gain: (2 to <4 kg/m²) | 1.01 (0.99 to 1.02) | 0.197 |
| Group e weight gain: (≥4 kg/m²) | 1.01 (0.99 to 1.03) | 0.230 |
| Type of delivery^§^ |  |  |
| Vaginal birth (reference group) | reference group | - |
| Instrumental delivery | 1.10 (1.07 to 1.12) | <0.001 |
| Caesarean section-emergency | 1.57 (1.54 to 1.59) | <0.001 |
| Caesarean section-elective | 1.53 (1.50 to 1.55) | <0.001 |
| Maternal age^§^ |  |  |
| 25-29 years (reference group) | reference group | - |
| 16-24 years | 1.01 (0.99 to 1.04) | 0.331 |
| 30-34 years | 1.00 (0.99 to 1.02) | 0.606 |
| ≥ 35 years | 1.01 (0.99 to 1.02) | 0.476 |
| Gestational age categories^§^ |  |  |
| Term (37 to 41 weeks) (reference group) | reference group | - |
| Pre-term (<37 weeks' gestation) | 1.17 (1.14 to 1.19) | <0.001 |
| Post-term (≥ 42 weeks) | 1.10 (0.98 to 1.24) | 0.103 |
| Interpregnancy (1^st^ birth to 2^nd^ birth) interval in months |  |  |
| <6 months (reference group) | reference group | - |
| 6-11 months | 0.94 (0.91 to 0.96) | <0.001 |
| 12-17 months | 0.96 (0.95 to 0.98) | <0.001 |
| 18-23 months | 0.99 (0.98 to 1.01) | 0.796 |
| 24-59 months | 1.01 (0.98 to 1.01) | <0.796 |
| ≥ 60 months | 0.99 (0.96 to 1.03) | 0.859 |
| Socio-economic disadvantage quintile |  |  |
| Quintile 5 (Least disadvantaged areas) (reference group) | reference group | - |
| Quintile 1 (Most disadvantaged areas) | 0.97 (0.96 to 0.99) | 0.002 |
| Quintile 2 | 0.99 (0.95 to 0.98) | 0.756 |
| Quintile 3 | 1.00 (0.98 to 1.01) | 0.947 |
| Quintile 4 | 0.98 (0.96 to 1.00) | 0.053 |

Note: ^§^Variable included for 2^nd^ birth only; β = a regression co-efficient using Generalised Linear Model (*GLM*) with a gamma family and log link function; Interpretation of exp (β): a one-unit increase in the predictor is associated with a [exp(β) −1]×100% increase or decrease in the expected maternal hospitalisation birthing costs; *CI* = confidence interval, reference category (considered as a low risk group); [*GDM* = gestational diabetes mellitus;](https://www.hopkinsmedicine.org/health/conditions-and-diseases/diabetes/gestational-diabetes) BMI = body mass index; p-value = the probability value; all variables were statistical significance at the 5% risk level in the crude model; ^€^The predictor variables were included in the adjusted model only if any label of the explanatory variable was significant at 5% or less risk level in the unadjusted model, which in turn was used to adjust for the effects of other potential confounders; ^€^Adjusted for *GDM* status, *BMI* change category, type of delivery, maternal age, gestational age in weeks categories, interpregnancy interval in months and socio-economic disadvantaged quintile.

Table S3.2 Bootstrapped sensitivity analysis for maternal hospital birthing costs in the second birth, using a multivariate generalised linear model with imputed values for missing BMI change

| Variables | Adjusted model for second birth^€^ | |
| --- | --- | --- |
|  | Exp (β) (95% CI) | p-value |
| GDM groups |  |  |
| No GDM in either pregnancy (reference group) | reference group |  |
| Diagnosed GDM in the first pregnancy but not in the second | 1.00 (0.97 to 1.02) | 0.604 |
| Diagnosed GDM in the second pregnancy but not in the first pregnancy | 1.07 (1.05 to 1.08) | <0.001 |
| Diagnosed GDM in both pregnancies | 1.09 (1.07 to 1.10) | <0.001 |
| BMI change quintile |  |  |
| Group b stable weight: ( -1 to <1 kg/m²) (reference group) | reference group |  |
| Group a weight loss: (<-1 kg/m²) | 1.01 (0.99 to 1.03) | 0.442 |
| Group c weight gain: (1 to <2 kg/m²) | 0.99 (0.97 to 1.00) | 0.183 |
| Group d weight gain: (2 to <4 kg/m²) | 1.01 (0.99 to 1.02) | 0.186 |
| Group e weight gain: (≥4 kg/m²) | 1.01 (0.99 to 1.03) | 0.263 |
| Type of delivery^§^ |  |  |
| Vaginal birth (reference group) | reference group |  |
| Instrumental delivery | 1.09 (1.06 to 1.13) | <0.001 |
| Caesarean section-emergency | 1.57 (1.54 to 1.61) | <0.001 |
| Caesarean section-elective | 1.52 (1.51 to 1.54) | <0.001 |
| Maternal age^§^ |  |  |
| 25-29 years (reference group) | reference group |  |
| 16-24 years | 1.01 (0.98 to 1.04) | 0.414 |
| 30-34 years | 1.00 (0.99 to 1.02) | 0.614 |
| ≥ 35 years | 1.01 (0.99 to 1.02) | 0.490 |
| Gestational age categories^§^ |  |  |
| Term (37 to 41 weeks) (reference group) | reference group |  |
| Pre-term (<37 weeks' gestation) | 1.17 (1.12 to 1.21) | <0.001 |
| Post-term (≥ 42 weeks) | 1.10 (1.01 to 1.20) | 0.034 |
| Interpregnancy (1^st^ birth to 2^nd^ birth) interval in months |  |  |
| <6 months (reference group) | reference group |  |
| 6-11 months | 0.93 (0.90 to 0.96) | <0.001 |
| 12-17 months | 0.96 (0.94 to 0.97) | <0.001 |
| 18-23 months | 0.99 (0.98 to 1.01) | 0.774 |
| 24-59 months | 1.01 (0.99 to 1.02) | 0.335 |
| ≥ 60 months | 0.99 (0.95 to 1.03) | 0.876 |
| Socio-economic disadvantage quintile |  |  |
| Quintile 5 (Least disadvantaged areas) (reference group) | reference group |  |
| Quintile 1 (Most disadvantaged areas) | 0.97 (0.96 to 0.99) | 0.002 |
| Quintile 2 | 0.99 (0.98 to 1.01) | 0.751 |
| Quintile 3 | 0.99 (0.98 to 1.01) | 0.947 |
| Quintile 4 | 0.98 (0.96 to 0.99) | 0.045 |

Note: ^§^Variable included for 2^nd^ birth only; β = a regression co-efficient using Generalised Linear Model with a gamma family and log link function; Interpretation of exp (β): a one-unit increase in the predictor is associated with a [exp(β) −1]×100% increase or decrease in the expected maternal hospitalisation birthing costs; *CI* = confidence interval, reference category (considered as a low risk group); [*GDM* = gestational diabetes mellitus;](https://www.hopkinsmedicine.org/health/conditions-and-diseases/diabetes/gestational-diabetes) BMI = body mass index; p-value = the probability value; all variables were statistical significance at the 5% risk level in the crude model; ^€^The predictor variables were included in the adjusted model only if any label of the explanatory variable was significant at 5% or less risk level in the unadjusted model, which in turn was used to adjust for the effects of other potential confounders; ^€^Adjusted for *GDM* status, *BMI* change category, type of delivery, maternal age, gestational age in weeks categories, interpregnancy interval in months and socio-economic disadvantaged quintil

Table S4. Interaction effect of gestational diabetes mellitus status and body mass index change on maternal hospital length of stay and medical birthing costs in the second birth

| Interaction between GDM status and BMI change category | Model for hospitalisation *length of stay* for maternal birthing in the second birth | | | | Model for maternal hospitalisation birthing *costs* in the second birth | | | |
| --- | --- | --- | --- | --- | --- | --- | --- | --- |
|  | Unadjusted model^£^ | | Adjusted model^€^ | | Unadjusted model^£^ | | Adjusted model^€^ | |
|  | Exp (β) (95% CI) | p-value | Exp (β) (95% CI) | p-value | Exp (β) (95% CI) | p-value | Exp (β) (95% CI) | p-value |
| GDM G1 × BMI change group b stable weight (-1 to <1 kg/m²) (reference group) | ref |  | ref |  | ref |  | ref |  |
| GDM G1 × BMI change group a weight loss (<-1 kg/m²) | 1.07 (1.01 to 1.14) | 0.017 | 1.05 (1.00 to 1.11) | 0.063 | 1.02 (1.01 to 1.05) | 0.026 | 1.01 (0.98 to 1.02) | 0.390 |
| GDM G1 × BMI change group c weight gain (1 to <2 kg/m²) | 1.04 (0.99 to 1.09) | 0.117 | 1.02 (0.98 to 1.05) | 0.316 | 1.01 (0.98 to 1.02) | 0.543 | 0.98 (0.97 to 1.00) | 0.171 |
| GDM G1 × BMI change group d weight gain (2 to <4 kg/m²) | 1.11 (1.06 to 1.16) | <0.001 | 1.03 (1.00 to 1.07) | 0.078 | 1.06 (1.04 to 1.08) | <0.001 | 1.01 (0.99 to 1.03) | 0.232 |
| GDM G1 × BMI change group e weight gain (≥4 kg/m²) | 1.16 (1.08 to 1.25) | <0.001 | 1.09 (1.03 to 1.15) | 0.002 | 1.05 (1.01 to 1.08) | 0.006 | 0.99 (0.96 to 1.02) | 0.894 |
| GDM G2 × BMI change group a weight loss (<-1 kg/m²) | 1.23 (1.06 to 1.42) | 0.006 | 1.13 (1.00 to 1.29) | 0.057 | 1.14 (1.05 to 1.24) | 0.001 | 1.07 (1.02 to 1.15) | 0.011 |
| GDM G2 × BMI change group b stable weight (-1 to <1 kg/m²) | 1.17 (1.09 to 1.26) | <0.001 | 1.12 (1.06 to 1.18) | <0.001 | 1.09 (1.05 to 1.13) | <0.001 | 1.05 (1.04 to 1.09) | <0.001 |
| GDM G2 × BMI change group c weight gain (1 to <2 kg/m²) | 1.44 (1.18 to 1.77) | <0.001 | 1.26 (1.13 to 1.40) | <0.001 | 1.11 (1.05 to 1.16) | <0.001 | 1.04 (1.01 to 1.07) | 0.004 |
| GDM G2 × BMI change group d weight gain (2 to <4 kg/m²) | 1.25 (1.15 to 1.36) | <0.001 | 1.18 (1.09 to 1.26) | <0.001 | 1.13 (1.09 to 1.18) | <0.001 | 1.07 (1.03 to 1.10) | <0.001 |
| GDM G2 × BMI change group e weight gain (≥4 kg/m²) | 1.52 (1.21 to 1.92) | <0.001 | 1.29 (1.15 to 1.46) | <0.001 | 1.20 (1.13 to 1.28) | <0.001 | 1.12 (1.06 to 1.19) | <0.001 |
| GDM G3 × BMI change group a weight loss (<-1 kg/m²) | 1.09 (0.98 to 1.22) | 0.106 | 1.04 (0.94 to 1.14) | 0.450 | 1.03 (0.96 to 1.10) | 0.417 | 0.98 (0.94 to 1.02) | 0.452 |
| GDM G3 × BMI change group b stable weight (-1 to <1 kg/m²) | 1.16 (1.07 to 1.26) | <0.001 | 1.13 (1.06 to 1.21) | <0.001 | 1.02 (0.98 to 1.07) | 0.240 | 1.04 (0.96 to 1.03) | 0.713 |
| GDM G3 × BMI change group c weight gain (1 to <2 kg/m²) | 1.29 (1.11 to 1.48) | 0.001 | 1.07 (0.95 to 1.21) | 0.268 | 1.15 (1.06 to 1.26) | 0.001 | 1.03 (0.98 to 1.10) | 0.203 |
| GDM G3 × BMI change group d weight gain (2 to <4 kg/m²) | 1.20 (1.06 to 1.36) | 0.003 | 1.10 (1.00 to 1.22) | 0.053 | 1.06 (0.99 to 1.15) | 0.086 | 0.98 (0.93 to 1.04) | 0.678 |
| GDM G3 × BMI change group e weight gain (≥4 kg/m²) | 1.43 (1.24 to 1.65) | <0.001 | 1.22 (1.06 to 1.41) | 0.006 | 1.16 (1.03 to 1.30) | 0.010 | 1.03 (0.95 to 1.12) | 0.357 |
| GDM G4 × BMI change group a weight loss (<-1 kg/m²) | 1.32 (1.18 to 1.48) | <0.001 | 1.25 (1.12 to 1.40) | <0.001 | 1.17 (1.08 to 1.26) | <0.001 | 1.09 (1.04 to 1.15) | <0.001 |
| GDM G4 × BMI change group b stable weight (-1 to <1 kg/m²) | 1.37 (1.25 to 1.50) | <0.001 | 1.25 (1.17 to 1.33) | <0.001 | 1.14 (1.09 to 1.18) | <0.001 | 1.08 (1.04 to 1.11) | <0.001 |
| GDM G4 × BMI change group c weight gain (1 to <2 kg/m²) | 1.40 (1.22 to 1.61) | <0.001 | 1.24 (1.10 to 1.39) | <0.001 | 1.16 (1.10 to 1.22) | <0.001 | 1.08 (1.04 to 1.12) | <0.001 |
| GDM G4 × BMI change group d weight gain (2 to <4 kg/m²) | 1.35 (1.24 to 1.47) | <0.001 | 1.21 (1.13 to 1.30) | <0.001 | 1.24 (1.17 to 1.31) | <0.001 | 1.12 (1.08 to 1.17) | <0.001 |
| GDM G4 × BMI change group e weight gain (≥4 kg/m²) | 1.51 (1.31 to 1.74) | <0.001 | 1.23 (1.12 to 1.36) | <0.001 | 1.24 (1.16 to 1.32) | <0.001 | 1.09 (1.04 to 1.15) | <0.001 |

GDM G1: Experienced no GDM in either pregnancy, GDM G2: Experienced no GDM in 1^st^ pregnancy but GDM in 2^nd^ pregnancy, GDM G3: Experienced GDM in 1^st^ pregnancy but no GDM in 2^nd^ pregnancy GDM G4: Experienced GDM in both pregnancies, Reference group = GDM G1 × BMI change group b stable weight (-1 to <1 kg/m²), ^§^Variable included for 2^nd^ birth only; β = a regression co-efficient using Generalised Linear Model (*GLM*) with a gamma family and log link function; Interpretation of exp (β): a one-unit increase in the predictor is associated with a [exp(β) −1]×100% increase or decrease in the expected length of stay (LOS) or hospitalisation birthing costs; *CI* = confidence interval, reference category (considered as a low risk group); [*GDM* = gestational diabetes mellitus;](https://www.hopkinsmedicine.org/health/conditions-and-diseases/diabetes/gestational-diabetes) BMI = body mass index; p-value = the probability value; all variables were statistical significance at the 5% risk level in the crude model; ^£^single variable was included in the unadjusted model only; ^€^The predictor variables were included in the adjusted model only if any label of the explanatory variable was significant at 5% or less risk level in the unadjusted model, which in turn was used to adjust for the effects of other potential confounders; ^€^Adjusted for *GDM* status, *BMI* change category, type of delivery, maternal age, gestational age in weeks categories, interpregnancy interval in months and socio-economic disadvantaged quintile.
